# Potential of Standard Perinatal Data for Measuring Violation of Birth Integrity

**DOI:** 10.3389/fgwh.2020.581244

**Published:** 2021-02-16

**Authors:** Céline Miani, Stephanie Batram-Zantvoort, Lisa Wandschneider, Jacob Spallek, Oliver Razum

**Affiliations:** ^1^Department of Epidemiology and International Public Health, School of Public Health, Bielefeld University, Bielefeld, Germany; ^2^Department of Public Health, Brandenburg University of Technology, Senftenberg, Germany

**Keywords:** childbirth, respectful maternity care, mistreatment, perinatal data, Germany, birth integrity, obstetric violence

## Abstract

**Background:** Measuring the phenomenon of violation of birth integrity (vBI) (e.g., obstetric violence) relies in part on the availability and content of maternity care providers' data. The population coverage and linkage possibilities that these data provide make for a yet untapped potential. Although vBI is a complex phenomenon best measured with dedicated instruments, we argue that maternity care providers' data could contribute to enhance our knowledge of the manifestations and frequency of vBI, and allow for analyses across different sub-groups of the population. Looking into the German standardized perinatal data, we investigate which variables are relevant to vBI-related research, and how complete their reporting is.

**Methods:** First, we analyse state-of-the-art frameworks and recommendations, and, for each vBI-related domain, we search for and list corresponding variables in the perinatal data which could contribute to a better understanding of vBI issues. Second, we use an example and analyse the content of perinatal data obtained between 2013 and 2016 in the context of the BaBi birth cohort study set in Bielefeld, Germany. We use descriptive statistics to assess the completeness of the data.

**Results:** The vBI-related variables can be classified in three main categories: discrimination based on specific patient socio-demographic attributes (e.g., height and weight to calculate BMI before pregnancy, foreign origin), indication for medical interventions (i.e., medicalization-related variables: indication for cesarean sections and induction), and supportive care, in particular the mobilization dimension (e.g., continuous fetal heartbeat monitoring). The data analyses included 876 births, of which 601 were vaginal birth. We found poor reporting on demographic variables in terms of completeness. Medicalization and mobilization variables are better documented, although limited in scope.

**Conclusions:** Putting more emphasis on the completeness of standardized data could increase their potential for vBI-related research. Perinatal data alone are insufficient to assess vBI, but a broader, theory-informed discussion of indicators to be included in standardized datasets would contribute to capturing the different aspects of integrity violation in a more systematic way and expand the evidence-base on different types of vBI.

## Background

There has been growing interest in recent years in the topic of obstetric violence, disrespect and abuse (D&A), mistreatment in childbirth, and respectful maternity care (RMC), all terms trying to capture some of the (violation of) integrity that one can face when giving birth (1–4). The multiple levels and dimensions of this phenomenon can better be encompassed in the concept of violation of birth integrity (vBI). We understand birth integrity as the mental and physical unity of a socially embodied human being striving to preserve their autonomy and dignity in pregnancy and childbirth. Birth integrity is a gender-inclusive concept, which aims to take into consideration the experiences of all birthing persons. A violation of birth integrity occurs when a birthing person's right to privacy is disregarded, self-determination is restricted, or dignity is violated. vBI can be both physical and emotional. Studies have shown that vBI is a very common phenomenon, and that in most settings the majority of women will experience at least a form of vBI during childbirth (5–10).

Several important reports and recommendations frame the research and practice with regard to vBI. For example, the WHO provides intrapartum care recommendations aiming to support positive childbirth experience (11). These recommendations are drawn from the latest knowledge of evidence-based practices in obstetrics and rely on the principles of the RMC charter which defines the universal rights of childbearing women (12). A large share of the academic literature also refers to the mistreatment framework of Bohren et al., a seven category framework which covers the main domains of vBI, from physical abuse to health systems conditions and constraints (1).

Reproductive health and childbirth in particular cannot be comprehended without taking into account gender relations, gender norms, and gender equality (13). The gender dimensions of vBI range from structural to relational and individual aspects. At the structural level, reproductive health has long been defined by the interrelated and historically grown processes of medicalization, pathologisation, and economization (14–16). The physiological processes of childbirth gets frequently interrupted through a highly medicalised organization of childbirth or a de-personalized view on laboring and childbearing women (17, 18). At the relational level, one of the most important gender dimensions of childbirth relates to the normalization of treating a woman in childbirth without respecting her autonomy, self-determination and integrity [e.g., by not asking for consent before performing a treatment or by failing to communicate important information about her health (19, 20)]. The delivery room can be seen as a mirror of societal attitudes that condone misogynic or discriminatory views upon women (21). Although these tendencies cannot be generalized, they must not be negated either as supported by the mounting evidence on vBI coming from countries all over the world (14–16, 18–23).

Research in the field of vBI during childbirth has so far taken different methodological approaches, including scales development, surveys, interviews, and direct observations (5, 24–27). All contribute to delineating the phenomenon and grasping the different aspects of it. However, one methodological aspect that has been so far underused or neglected is the possibility to capture some of the vBI phenomenon through standardized medical records. Indeed, from an epidemiological perspective, measuring the phenomenon of vBI in childbirth relies in part on the availability and completeness of child-birth related medical records, also called hospital (or other maternity care providers') data, or, as in Germany where our study is set, perinatal data. The population coverage provided by such data, as well as linkage possibilities with many information systems, makes for a yet untapped potential.

Although vBI is a complex phenomenon that can only be fully grasped with specifically designed instruments and the plurality of points of views (i.e., providers, women giving birth, and observers), we argue that some of the variables in the perinatal data could contribute to enhance our knowledge of the manifestations and frequency of vBI, and allow for analyses across different sub-groups of the population.

In the following, we propose to assess the potential of the German perinatal data for capturing aspects of vBI. We aim to answer the following questions:

- Which variables pertaining to the different dimensions of vBI are included in the data?
- To which extent are those variables complete?
- What could be done to improve the potential of perinatal data in capturing vBI?

## Methods

In order to assess the potential of perinatal data for measuring vBI during childbirth, we perform the following steps.

First, to identify which perinatal data variables pertain to the dimensions of vBI described in the literature, we analyse state-of-the-art frameworks and recommendations, namely the mistreatment framework by Bohren et al. (1, 24), the RMC Charter (12) and the WHO recommendations for evidenced-based care in childbirth (11). For each vBI-related domain and dimension of evidenced-based care during childbirth, we search for and list corresponding variables in the perinatal data which could contribute to a better understanding of vBI issues. Our attribution of variables to dimensions is based on the information included in the frameworks, on variables already used in the scientific literature to implement those frameworks [e.g., Bohren et al. (24); Montesinos-Segura et al. (25); Afulani et al. (26)] and on our own judgement. Through this process, we are also able to identify indicators which are referred to in the frameworks but currently not included in routine perinatal data sets. In Germany, data about all hospital births are collected in a standardized way and annually reported by the Institute for Quality Assurance and Transparency in Healthcare (*Institut für Qualitätssicherung und Transparenz im Gesundheitswesen*, IQTIG), also in charge of validation and anonymization of the data. The 2020 perinatal data variable list can be found in the documentation of the IQTIG (28). Perinatal data have a similar structure throughout the country; they are standardized records which include information about the mother, the pregnancy, the birth, and the birth outcomes for both the mother and the infant.

Second, to assess to which extent these data can be complete, and therefore useful for vBI-research, we use an example and analyse the content of perinatal data obtained between 2013 and 2016 in the context of the BaBi birth cohort study set in Bielefeld (350,000 inhabitants), North-Rhine Westphalia, Germany (29). The data were provided by the main maternity care providers (*n* = 5) in Bielefeld for each participant recruited in the cohort study. Only in-hospital births are included in the present study. The BaBi study was approved by the ethical committee of the Medical Faculty of Muenster University and the Data Protection Board of Bielefeld University. We use descriptive statistics to assess the completeness of the data. Analyses are performed with SAS 9.4.

## Results

### vBI Research-Relevant Variables in the Perinatal Data

Table 1 lists the domains of vBI described in previous frameworks and recommendations and specifies how some of the variables from the perinatal data relate to them. The last column also highlights variables that would fall into one of the vBI domains but are not included in the perinatal data. The vBI-related variables can be classified in three main categories: discrimination based on specific socio-demographic attributes of the birthing person (e.g., height and weight to calculate BMI before pregnancy, pre-existing conditions), indication for medical interventions (i.e., medicalization-related variables), and supportive care, in particular the mobilization dimension (e.g., continuous monitoring during labor). Other dimensions of vBI, i.e., physical, sexual or verbal abuse, and health system constraints were considered not suited for investigation through perinatal data or equivalent.

**Table 1:** Dimensions of vBI and corresponding vBI-related variables of interest that are included in, or missing from, the perinatal data.

**Dimensions of vBI**	**vBI-related variables included in the perinatal data [2020 version (28)]**	**vBI-related variables not included in the perinatal data**
**Discrimination:**		
The RMC charter states that every women has “the right to equality, freedom from discrimination and equitable care” (Art. V) (12). Stigma and discrimination also constitute a dimension of the mistreatment typology, encompassing the discriminative care of childbearing women based on specific patient attributes or medical conditions (1).	Variables on socioeconomic background (postcode as an ecological proxy for individual socioeconomic status, social or economic difficulties perceived as a risk factor during pregnancy). Variables on physical characteristics (height, weight), age, health-related behavior (drug abuse, mother's lack of cooperation, discharge diagnosis: discharge against medical advice). Variables on pre-existing conditions: diabetes, heart disease, mental health issues, etc. Pregnancies' history: number of previous pregnancies, number of previous live births, having had more than two abortions or miscarriages. Child-related variables: malformations, morbidity and weight.	Variables on socioeconomic background: migration history^*^ (Germany as a country of origin and, for foreigners, region of origin), or marital status^*^ (i.e., single mothers), employment status^*^, occupation^*^. Health-related variables: e.g., number of abortions^*^, number of miscarriages^*^, smoking status (cigarettes per day)^*^. Language/communication related variables: woman's ability to speak and understand language offered at hospital, presence of translator to support communication between health personal and woman. Further differentiation of special needs in childbirth: experience of sexual violence, experience of domestic violence, refugee experience, prior (birth) trauma, cognitive impairments, physical disability. Gender identity and sexual orientation variables: transgender, non-binary, in a non-heteronormative relationship. If the child will be given up for adoption or child taken into state care.
**Medicalization:**		
According to the RMC charter, every woman has the right to healthcare and to the highest attainable level of health (Art. VI) (12). This includes the provision of evidence-based care practices and the prevention of over- or under-medicalization in intrapartum care, as stated in the WHO recommendations on positive childbirth experience: “There has been a substantial increase over the last two decades in the application of a range of labor practices to initiate, accelerate, terminate, regulate or monitor the physiological process of labor, with the aim of improving outcomes for women and babies. This increasing medicalization of childbirth processes tends to undermine the woman's own capability to give birth and negatively impacts her childbirth experience” (11). Therefore, clear, evidence-based indications reasoning the application of clinical intervention in labor or birth are essential to prevent (over-) medicalization.	Indication for cesarean section.	Indications for the use of other obstetric interventions, e.g., labor induction^*^, augmentation of labor (oxytocin, other), tocolysis, cardiotocography (CTG –fetal heartbeat recording)/ continuous CTG, episiotomy, vaginal operations.
**Supportive Care:**		
According to the RMC charter, every woman has the right to liberty, autonomy, self-determination, and freedom from coercion (Art. VII) as well as “(…) respect for her choices and preferences” (Art. II) (12). Within the mistreatment framework, the denial of mobility and a lack of respect for a woman's preferred birth position contributes to loss of autonomy and is a reflection of poor rapport between the woman and the care providers (1). According to the WHO recommendations, supportive care includes enabling women to move around freely during labor. In this respect, routine CTG on labor admission is not recommended for healthy pregnant women (instead: Doppler ultrasound), neither is continuous CTG during spontaneous labor. Encouraging women (with and without epidural analgesia) to a birth position of their individual choice, including upright positions, is also recommended (11). Other aspects reflecting supportive care comprise effective communication (language), the presence of birth companions, the provision of pain relief, skin-to-skin contact of mothers and newborns during the 1st h of birth and support of breastfeeding.	Variables on interventions that may hinder mobilization: CTG on admission, continuous CTG (internal or external).	Birth position^*^ as a proxy for mobilization, doppler ultrasound^*^. Other supportive measures: language offered, skin-to-skin contact with newborn, availability of breastfeeding support, birth companion present during labor and birth.

* *Denotes variables documented in the perinatal data until 2017, and removed afterwards [see for example the 2015 variable list, then curated by the aQua Institute (30)]*.

### Completeness of vBI-Relevant Variables in the Perinatal Data

We include 876 births in our analyses. The number of births per facility ranges from 20 to 340. There are 601 vaginal births vs. 275 cesarean sections. Table 2 shows the completeness in percent across providers (minimum, maximum and mean completeness) of a selection of vBI-related variables, i.e., those for which a value was required for all participants or a specific group of participants (e.g., participants who had a cesarean section). The last column shows the percentage of cases for which a value has been attributed.

**Table 2:** Completeness (%) of vBI-related variables across providers and total percentage of reported values.

	**% reported values by provider^a^**		**% reported values, all providers included**
**Dimensions and variables**	**Min**	**Max**	
**Discrimination:**			
Postcode	70,6	100,0	88,2
Height	85,0	100,0	99,7
Weight at the beginning of pregnancy	6,8	100,0	73,9
Prior live births	95,0	100,0	99,9
Child outcomes:			
Stillbirth	75,0	100,0	99,2
Weight	99,41	100	99,8
Length	97,39	100	98,7
Malformation	55,88	100	71,0
Malformation prenatal	14,69	99,41	64,4
**Medicalization:**			
Indication for cesarean section^*^	85,7	100,0	96,7
**Supportive care:**			
CTG on admission^**^	33,3	100,0	86,0
Continuous CTG monitoring^**^	14,3	66,2	44,4
Continuous external CTG monitoring^***^	75,8	100,0	88,0
Continuous internal CTG monitoring^***^	69,7	100	85,4

a *Data protection agreements do not allow comparing providers to each other. We therefore refrain from publishing percentage of reported values for each provider and show instead the lowest and highest completeness rates among the five providers, as well as the percentage of reported values across the whole sample*.

* *Denotes a variable for which completeness was calculated among the cesarean section births only*.

** *Denotes a variable for which completeness was calculated among the vaginal births only*.

*** *Denotes a variable for which completeness was calculated among the cases for which the value for “continuous CTG monitoring” was “yes”*.

With regard to discrimination-related variables, completeness varies depending on indicators and across the care facilities. The values for height of the woman, prior live births, still birth, and weight and length of the newborn are only missing in a couple of cases. Weight of the woman at the beginning of pregnancy is not as well-documented, limiting the possibility to include pre-pregnancy BMI into analyses. In the medicalization dimension, indication for induction for cesarean section is relatively well-documented, with an indication for cesarean section being reported for 96.7% of the cesarean section births. In the supportive care dimension, monitoring through CTG is unequally documented across providers. However, the type of CTG (external or internal) for participants who received a control CTG is documented in more than 85% of the cases.

## Discussion

Standardized perinatal data can be helpful in providing contextual information relating to the birth, details about the birth process and the type of care that was given, as well as the distribution of birth-related events across different patient groups. In this study, we find suboptimal reporting on demographic variables in terms of scope and completeness. The medicalization- and mobilization-related variables are better documented, although limited in scope.

We can only hypothesize why the data is not complete, since we have received no information from providers that would explain it. Completeness of the data is dependent on time constraints, priorities and can be reflected in some built-in functions within information systems (e.g., mandatory fields) (31). Constraints and priorities can be set at the level of the individual who enters the values for each variable, but also the level of the organization (e.g., priority given to a set of performance indicators) (32) and on a more cultural level (e.g., position at birth very important to midwives, not that important to doctors, to simplify). All these levels are relevant in a gender analysis of childbirth care. Gender norms not only shape the experiences of women, and the relational interactions between women and providers, but also the systems that are in place and the structures that shape obstetric care (33). Next, we will interview maternity care providers to understand their data collection constraints and priorities and potential reporting bias in real-life settings.

Incorrect or missing values can also be due to human error or technical issues, especially when different information systems (e.g., software) and sources are involved in the handling of data. For example, in the perinatal data, indicators of risk pregnancies are entered based on the maternity booklet kept by the pregnant woman, in which either the gynecologist or the midwife documents their assessments throughout pregnancy. This introduces additional reporting layers and constraints, and more opportunities to incorrectly enter values. Also, different versions of the perinatal data have been used over the years, and it may create discrepancies and linkage issues.

By nature, the perinatal data comprise, for the majority, medical variables, measures, and indicators that have the advantage of being standardized internationally (e.g., Apgar score). The data collected in hospitals has some medical relevance for the individual patient and respond to needs for organizational monitoring, and beyond, population health assessment and epidemiological endeavors [e.g., (34, 35)]. One could envisage another dimension, theoretically and ethically grounded, which would be to contribute to equity and protection of birth integrity. For example, one could consider including a variable specifying if the woman's companion of choice attended the birth (an aspect of supportive care), and if this was not authorized, why. The position at birth for vaginal deliveries is also a variable that has potential in terms of evidence-based practice, and yet is not prioritized. It has been excluded from the perinatal dataset in 2017 in Germany (30, 36), although it is still documented for out-of-hospital births. It is also integrity-relevant to reflect on some interventions which are likely to be performed, but not documented in the first place. This is the case for example of fundal pressure (also called “Kristeller maneuver”). Studies on vBI have found that it is a frequently used procedure although there is no evidence of its benefit. Instead, it carries high vBI potential and the WHO recommendations strongly discourage it due to its potential for harm (11, 37–39).

The scope of perinatal data in Germany is increasingly focused on risk reporting and medical outcomes. In 2017, several socio-demographic variables that could inform discrimination analyses (e.g., migration background, occupational and marital status) and variables relevant to supportive care and medicalization were dropped from the perinatal data reporting (30, 36). This is in contradiction with the recommendations of the Euro-PERISTAT project on monitoring of perinatal health which includes indicators on populations' characteristics (40).

The potential for intersectional analyses, which would investigate the interaction of different sources of disadvantage or discrimination (41), is furthermore impeded by the limited and incomplete collection of socio-demographic variables. A couple of markers of risk pregnancy relating to drug abuse and social and economic deprivation, as well as the reporting of pre-existing medical conditions, have the potential to identify some of the most vulnerable individuals. However, any vBI research looking to investigate the link between disability, racism, xenophobia, and vBI would not be able to do so with the limited information comprised in the perinatal data. The potential of medical records for intersectional analyses is rarely investigated in population health research. First approaches using Swedish register data have highlighted complex patterns of different chronic diseases across intersectional strata, defined by e.g., sex, age, civil- and migration status, education, and income (42–45). Medical records provide large databases with sufficient statistical power, which is an indispensable feature in quantitative intersectional analyses to obtain precise estimates (45). The precise mapping of socioeconomic health disparities would allow to identify relevant at-risk sub-populations and allocate targeted prevention or intervention resources (43).

Perinatal data alone cannot be enough to assess vBI in childbirth, for several reasons: independently of how good and complete they are, they will only provide the view of the care provider, and it is of the utmost importance to also seek the view of the women. Studies have shown discrepancies between what women report and what is observed by researchers (6, 9, 24, 46). Discrepancies certainly exist also between the views of the women and the ones of the providers as objectified in the inputting of perinatal data. Another reason is that some of these variables lack explanatory power. Knowing just that there was an indication for induction is not that meaningful in itself. It has to relate to other variables and other vectors of experience.

However, despite these caveats, there could be potential in the use of perinatal data for vBI research, at least in Germany. It would imply using an already existing information system to address the knowledge gaps with regard to a “new” or rather emerging epidemiological topic. But it would also mean, and this is not a simple task, investing in improving the completeness of the data, and in reviewing –or adding a few indicators. Medical records have long been used to monitor progress in terms of maternal health (47). However, to date, population-based data initiatives on vBI are missing in most countries. Research could then address this gap through qualitative studies involving maternity care providers and data quality assurance managers in order (i) to understand the constraints and challenges of collecting and recording data; and (ii) to investigate the potential for changes in the selection of variables and the prioritization of themes.

## Strengths and Limitations

This study is to our knowledge the first to adopt a gender lens to look at the potential of perinatal data for vBI research. The selection of variables was guided by state-of-the-art frameworks [e.g., the mistreatment framework of Bohren et al. (1)], allowing for a reflection on evidence-based practice and the implementation of safe and respectful reproductive health services. A limitation of the study is that the number of births included is relatively small, and the generalisability of the study is limited. Finally, the study is exploratory; it does not aim at systematically assessing the completeness of all vBI-related variables. It is rather a critical and theory-informed look at the perinatal data for capturing vBI-related experiences.

## Conclusions

Putting more emphasis on the completeness of standardized data could increase their potential for vBI-related research. Perinatal data alone are insufficient to assess vBI in childbirth. A broader, theory-informed discussion of indicators to be included in standardized datasets would contribute to capturing aspects of integrity violation in a more systematic way and expand the evidence-base on different types of vBI.

## Summary Table

*What was already known on the topic:*

- Violation of birth integrity (vBI) manifestations are increasingly documented in the literature, but barely through the use of perinatal data (which are based on medical records).
- vBI includes several dimensions, such as discrimination based on personal attributes, medicalization, and mobilization.

*What this study added to our knowledge:*

- This study brings a new perspective on vBI, applying a gender lens to look at the potential of perinatal data for vBI research.
- There are variables in the perinatal data that could contribute to vBI research, e.g., about medicalization and supportive care, although perinatal data alone cannot be enough to assess vBI in childbirth.
- A broader, theory-informed discussion of indicators to be included in standardized records would contribute to capturing aspects of integrity violation in a more systematic way and reinforce the evidence-base on different types of vBI.

## Data Availability Statement

The data analyzed in this study is subject to the following licenses/restrictions: Data are available upon request due to ethical restrictions, which pertain to the data protection agreements that are in place between the providers who shared the data, the study participants, and the research team, and which fall under the European Union data protection regulation. Interested researchers may submit requests to Dr. Céline Miani, leader of the BaBi Study, School of Public Health, Bielefeld University. Contact: Universitätsstraße 25, 33615 Bielefeld, Germany. E-mail: celine.miani@uni-bielefeld.de.

## Ethics Statement

The BaBi study was approved by the ethical committee of the Medical Faculty of Muenster University and the Data Protection Board of Bielefeld University. The participants provided their written informed consent to participate in this study.

## Author Contributions

CM conceptualized the study with SB-Z. CM, SB-Z, and LW conducted the data analyses and wrote the first draft of the paper. OR provided substantial feedback on different versions of the manuscript. JS designed the BaBi-study and was its original PI, together with OR. All authors contributed to the final draft.

## Conflict of Interest

The authors declare that the research was conducted in the absence of any commercial or financial relationships that could be construed as a potential conflict of interest.

## Abbreviations

CTG: Cardiotocography
D&A: Disrespect and Abuse
RMC: Respectful maternity care
vBI: Violation of birth integrity
WHO: World Health Organization.
